# Isolation, identification and pathogenicity of local entomopathogenic bacteria as biological control agents against the wild cochineal *Dactylopius opuntiae* (Cockerell) on cactus pear in Morocco

**DOI:** 10.1038/s41598-023-48976-8

**Published:** 2023-12-08

**Authors:** Karim El Fakhouri, Chaimae Ramdani, Abderrahim Aasfar, Rachid Boulamtat, Badreddine Sijilmassi, Mustapha El Bouhssini, Issam Meftah Kadmiri

**Affiliations:** 1 AgroBioSciences Program, College of Agriculture and Environmental Science, Mohammed VI Polytechnic University, Lot 660, Hay Moulay Rachid, 43150 Benguerir, Morocco; 2 Entomology Laboratory, International Center for Agricultural Research in the Dry Areas (ICARDA), Rabat Institutes, P.O. Box 6299, Rabat, Morocco; 3 Plant and Microbial Biotechnology center, Moroccan Foundation for Advanced Science, Innovation and Rescarch (MAScIR), Mohammed VI Polytechnic University, Lot 660, Hay Moulay Rachid, 43150, Benguerir, Morocco; 4 Rhizobium Laboratory, Genetic Resources Section, International Center for Agricultural Research in the Dry Areas (ICARDA), Rabat Institutes, P.O. Box 6299, Rabat, Morocco

**Keywords:** Microbiology, Plant sciences, Ecology

## Abstract

The *Opuntia ficus-indica* (L.) cactus, a crucial crop in Morocco, is threatened by the wild cochineal, *Dactylopius opuntiae* (Cockerell). The aim of this research was to investigate the efficacy of nine bacterial strains against both *D. opuntiae* nymphs and adults females applied individually or after black soap in the laboratory, greenhouse, and field conditions. Using the partial 16S ribosomal DNA, the bacterial isolates were identified as *Pseudomonas koreensis*, *Pseudomonas* sp., *Burkholderia* sp. and *Bacillus* sp. Under laboratory conditions, the insecticidal activity of *P. koreensis* strain 66Ms.04 showed the level mortality (88%) of adult females’ at 10^8^ CFU/mL, 7 days after application. At a concentration of 10^8^ CFU/mL, *P. koreensis* strain 66Ms.04 and *Pseudomonas* sp. (strains 37 and 5) caused 100% nymphs mortality rate three days after application. Under greenhouse conditions, the use of *P. koreensis* strain 66Ms.04 at 10^8^ CFU/mL following the application of black soap (60 g/L) demonstrated the maximum levels of females and nymphs’ mortalities with 80 and 91.25%, respectively, after 8 days of treatment. In field conditions, the combined application of the *P. koreensis* strain 66Ms.04 at 10^8^ CFU/mL with black soap at 60 g/L, for an interval of 7 days, significantly increased the mortality of adult females to 93.33% at 7 days after the second application. These findings showed that the combined treatment of *P. koreensis* strain 66Ms.04 with black soap can be a potent and eco-friendly pesticide against *D. opuntiae.*

## Introduction

*Opuntia ficus-indica* (L.) Mill. (Caryophyllales: Cactaceae) commonly called prickly pear or nopal cactus, belongs to the dicotyledonous angiosperm family Cactaceae and originates from Mexico. This species has the ability to thrive in arid and semi-arid environments and geographically distributed in South Africa, Latin America, and the Mediterranean countries^1^. It has special adaptive mechanisms and a high biomass production capacity, which allows it to grow in adverse conditions, such as high temperatures and nutritionally poor soils subject to erosion^2^. The cactus has been present in Morocco since 1770 and it’s currently widely distributed in the national landscape. As a result of drought, the cactus area has expanded significantly over the past twenty years, it has increased from about 50,000 ha in 1998 to more than 150,000 ha in 2017^3^. Cactus pear is considered as great source of food and feed. It has been traditionally recognized as a nutrient that can provide valuable health benefits, in addition to its wide range of uses in the pharmaceutical industry^4^. The modernization of Moroccan agriculture (Green Generation Plan) encourages cactus plantation as an alternative crop in less favorable regions^3^. However, the crop has been suffering from the attack of a sap-sucking insect pest, the wild cochineal *Dactylopius opuntiae* (Hemiptera: Dactylopiidae) since 2014. The rapid and aggressive spread of the cochineal pest in various regions of Morocco has led to significant socio-economic consequences^5^. This pest is widely distributed throughout the Mediterranean basin and has become a serious threat to the prickly-pear crop^6^. According to Ochoa et al.^7^, *D. opuntiae* is a pest that is present in more than 30 countries where cactus is cultivated. Both nymphs and adult females of *D. opuntiae* suck sap from the cladodes of the plants, leading to their desiccation, weakening, and death^8^. The females of the wild cochineal have a white waxy coating that provides a physical barrier against predators and helps them to maintain an ideal moisture level.

Significant progress has been achieved in applying of the integrated pest management approach using a combination of techniques such as planting cochineal-resistant host plants, biological control and the use of biopesticides derived from natural sources to effectively manage the wild cochineal population in Morocco^5, 9–11^. Over the last two decades, the use of synthetic chemicals has led to several environmental problems and health risks^12^. Some chemical pesticides have already been banned by the EU and the US due to environmental and human health problems^13^.

Chemical insecticides have played a major role in the control of insect pests. However, the growing demand to reduce synthetic chemicals use due to environmental and human health concerns, in addition to pesticide resistance issues, is fuelling interest in innovative and sustainable approaches to manage this new invasive cochineal *D. opuntiae*.

Biological control with entomopathogenic fungi^14^ and entomopathogenic bacteria^15^ offers a better alternative to synthetic chemical pesticides, because of the high specificity of the biopesticides, their easy biodegradability, their short shelf-life and environmental friendly for sustainable agriculture^16, 17^. Many microbial pathogens of insects are intensively investigated to develop environmentally friendly pest management strategies in agriculture^18^. Over 100 bacterial species with entomopathogenic activity have been identified as both exo- and endo-pathogens of arthropods^18, 19^. But only some of these bacterial entomopathogens are commercially available for agricultural uses. Some of the bacteria used commercially are: *Bacillus thuringiensis, Bacillus Lysinibacillus, Bacillus popilliae, Pseudomonas alcaligenes, Clostridium bifermentans, Saccharopolyspora spinosa, Pseudomonas aureofaciens, Streptomyces avermitilis* and *Serratia entomophila* were the most studied^20–22^. In a previous research, Idris et al.^15^ showed the potential of crude enzymes produced by *Bacillus subtilis*, the local strain SY134D, to control *D. opuntiae* insects under laboratory conditions.

The aim of the present investigation is to study the insecticidal effect of different bacterial strains isolated from Moroccan soils for the management of nymphs and females of *D. opuntiae* in the laboratory, greenhouse, and field conditions. The findings of this study will be exploited in the development of microbial insecticide formulation, which can effectively protect the prickly pear from the scale insect *D. opuntiae* as an eco-friendly, target‐specific, easily biodegradable, and safer alternative agricultural product.

## Materials and methods

### Isolation of the bacterial strains

The bacterial strains used in this work were isolated from a set of soils belonging to different regions of Morocco. Table 1 shows the geographical locations and site details of sampled soils. The soil samples were collected at a depth of 30 cm close to the roots. The soils were placed in sterile polypropylene bags and immediately transported to the laboratory. The soil samples were stored at 4°C and processed within 48 h by examining 0.1 g of a subsample from each sample. The subsamples were transferred and homogenized in 1 mL of sterile physiological water. Soil suspensions were serially diluted (from 10^–2^ to10^–9^) and aliquots were placed on Burk's agar plates using a 100 µL spreader^23^, then incubated for 4–5 days at 30 °C. After that, single colonies were preserved in Burk's agar medium for additional purification. For long-term storage, each isolate was kept at -80°C in liquid Burk's medium, which contains 30% (v/v) glycerol.

**Table 1:** Geographical locations and site details of sampled soils.

Code	Area	Location name	Latitude	Longitude	Collection date	Crop system
S1	El Haouz	Ait Ourir	31.5801	− 7.655	16-02-2018	Faba bean
S2	El Haouz	Tamazouzte	31.5801	− 7.655	16-02-2018	Turnip
S3	TAZA	Merzouka	34.2138	− 4.1205	24-01-2018	Chickpea
S4	El Jadida–Bennour	Sidi Ismail	33.059	− 8.4349	12-01-2018	Faba bean
S5	El Jadida–Sidi Bennour	Haouzia	33.1504	− 8.478	12-01-2018	Zea mays

### Identification of the bacterial strains

The isolated bacterial strains were identified based on partial 16S ribosomal DNA (16S rDNA). The genomic DNA of bacterial strains was extracted by Pure- Link™ Genomic DNA Mini Kit (Invirogen, K182001). PCR reactions were performed using DreamTaq DNA Polymerase PCR Master Mix comprising 1 μg DNA, 0.4 mM dNTPs, 4 mM MgCl2 (Invirogen, K1071), and 1 μM of each of the following primers 27F 5′-AGA GTT TGA TCC TGG CTC AG-3′/1492R 5′- ACG GTT ACC TTG TTA CGA CTT-3′^24^were used to amplify the 16S rDNA, in a final reaction volume of 25 μL. The process of thermocycling involved subjecting the sample to different temperatures in a PCR machine. The first step was DNA denaturation at 95 °C for 1 min, followed by the annealing with 35 cycles of 95 °C for 30 s, 53 °C for 30 s, and 72 °C for 1 min. Finally, the sample was subjected to a terminal extension at 72 °C for 15 min. The resulting PCR products were verified using a 1% agarose gel and purified using the PureLink Quick Gel Extraction Kit from Invitrogen (K220001). The PCR products were then transferred to Secugen S.L. (https://www.secugen.es) for sequencing, and the obtained sequences were compared with those available in the NCBI server (https://blast.ncbi.nlm.nih.gov/Blast.cgi)^25^ and submitted in GenBank (Table 2). To determine the evolutionary relationships between the sequences, a phylogenetic tree was constructed using the neighbor-joining method in the MEGA 7.0 software.

**Table 2:** Microbial isolates identified by 16S rDNA sequence with their percentage similarity.

Isolate ID	Isolation site	Genbank accession no	Related species	Accession no	Similarity (%)
5Ms	S1	ON754230	*Pseudomonas* sp*.*	NR_025228.1	96.52
18Ms	S2	ON754236	*Burkholderia* sp.	NR_104978.1	99.5
27Ms	S1	ON754242	*Pseudomonas* sp.	NR_025228.1	98.52
37Ms	S3	ON754243	*Pseudomonas* sp.	NR_025228.1	99.68
38Ms	S4	–	–	–	–
41Ms	S5	ON754244	*Bacillus* sp*.*	NR_148786.1	98.7
66Ms.04	S3	ON668306	*Pseudomonas koreensis*	NR_025228.1	99.79
87Ms	S3	ON754246	*Pseudomonas* sp.	NR_025228.1	99.68

### Preparation of bacterial suspensions

The bacterial stock was initiated from a single colony of each of the eight bacteria, inoculated in liquid media Luria–Bertani Broth (LB), and grown for 48 h at 25 ± 2 °C in darkness under agitation at 150 rpm. The optical densities of bacterial suspensions were determined using UV/VIS spectrophotometer (T80, PG-Instruments) and the populations of cells at the various optical densities were determined by dilution plating.

### Laboratory bioassays

#### Insect rearing

One-year-old healthy young cladodes of *O. ficus-indica*, were planted in plastic pots measuring 27 cm in diameter and 24 cm in height. The pots were filled with a soil mixture consisting of equal parts of sand, peat, and soil, with a volume ratio of 1:1:1. Healthy young cladodes were first placed in a glasshouse at 30 ± 5°C where they were subjected to a heavy infestation of cladodes collected from the Rabat region of Morocco (33°59′57″ N 6°23′27″ W). All cactus cladodes used in the trials, were collected in conformity with Moroccan Agriculture Ministry guidelines and regulations. Each cladode previously infested with the *D. opuniae* was placed in two pots to infest the healthy cladodes. After exposing the cladodes to the infested colonies for a month, the colonies with adult females were selected to be used in different experiments.

#### Contact toxicity

The immersion application method was used to assess the contact toxicity of eight bacterial strains. The study was carried out in the laboratory under controlled conditions of 24 ± 2 °C temperature, 75% humidity, and a 14:10 (light:dark) photoperiod. Three concentrations (10^6^, 10^7^ and 10^8^ CFU/mL) were specifically selected on the basis of preliminary tests, and were used mixing the bacterial strains with water.

#### Adult females of *D. opuntiae*

Ten first instar–mature females *D. opuntiae* of the same age were immersed in different bacterial strains at different concentrations for five seconds and deposited separately, using an entomological brush, on cladodes of the same size placed in Petri dishes (9 cm in diameter). The control adult females were immersed in water. The experiments were performed using a completely randomized design (CRD) with five replicates. The number of dead adult females was recorded every 24 h for a period of 8 days after the use of various treatments, using a binocular microscope (MoticDM-143). The dead females showed a dark brown color, and their bodies were desiccated.

#### Nymphs of *D. opuntiae*

Ten first instar nymphs of *D. opuntiae* of the same age (21 h) were deposited on cladodes of the same size placed in Petri dishes and were directly sprayed with different bacterial strains of different concentrations. The control nymphs were sprayed with distilled water. The bioassays were done using a completely randomized design (CRD) with five replicates. Mortality of nymphs was recorded every 24 h for a period of 8 days. The dead nymphs showed no movement and had dye modifications.

#### Toxicity of different bacterial strains alone or in combination with Black Soap under greenhouse conditions

The insecticidal activity of four bacterial strains (the most effective bacterial strains selected from laboratory tests) was tested alone or in combination with the black soap by contact application. The bacterial strains were used at a concentration of 10^8^ CFU/Ml, while black soap with a concentration of 60 g/L was applied to facilitate the degradation of cuticular wax^5^ and then exposed the females to different bacterial strains. The bioassays were conducted in a completely randomized design (CRD) with four repetitions. The experimental procedure involved the application of the soap solution to cladodes first, followed by the application of bacterial strains using a 1L hand sprayer. Mortality rates of nymphs and females were recorded every 24 h for a period of 8 days after treatment. The application was decided at the medium level of infestation (26–50%) using Silva`s modified rating scale^26^ to determine the severity of wild scale infestation in cactus pear plants as follows: 0—not infested 0%; 1—low infestation 1–25%; 2—medium infestation 26–50%; 3—high infestation of 51–75%; 4—extensive infestation of 76–100%.

### Field bioassay

The bacterial strains that exhibited considerable toxicity against nymphs and females of *D. opuntiae* in the laboratory and greenhouse conditions were chosen to evaluate their effectiveness in the field conditions from October to November 2021.

The field experiment was carried out near Rabat region, Morocco (33°59′57″ N 6°23′27″ W). The experimental design adopted a randomized complete block with each treatment repeated three times. In each plot, three cladodes were selected for treatment. The treatments consisted of applying the *P. koreensis* strain 66Ms.04 at a concentration of 10^8^ CFU/mL either alone on the cladodes, or on cladodes that had been previously sprayed with black soap at a concentration of 60 g/L which served to remove the cuticular wax and exposed the females and nymphs to the used bacterial strain. Two different controls were used, the first control is cladodes treated with water only, and the second control is cladodes treated with black soap at 60g/L. The tested bacterial strain solutions were combined with the 0.01% of Triton X-100-stabilized emulsion used to improve the solubility and dispersion of the bacteria with water before application using a 2L hand sprayer, with a 250 l/ha rate and 8 ml min^−1^ frequency. The second spray was done seven days after the first one. Mortality of nymphs and adult females was recorded 3, 5 and 7 days of the first and second sprays. The application was decided at the low to medium levels of (26–50%) infestation using Silva`s modified rating scale (1991).

### Statistical analysis

Before performing statistical analysis, mortality percentages were transformed into angular values (arcsine √P). In the laboratory, the transformed percentages were analysed using a two-way analysis of variance (ANOVA) to investigate the effects of bacterial strain concentrations and bacterial strain source. In order to estimate the lethal concentration for 50 and 90% mortality (LC_50_ and LC_90_ respectively), intercept, slope of the regression line, and fiducial limits, concentration-mortality, data was subjected to probit analysis^27^ using IBM SPSS Statistics 27.0. In the greenhouse and field experiments, the transformed percentages were subjected to a one-way ANOVA. To compare means, Tukey’s test was employed at a significance level of *P* < 0.05. All statistical analyses were conducted using Genstat (21st Edition, VSN International, Hemel Hempstead, UK).

## Results

### Identification of the bacterial strains

**Figure 1:**
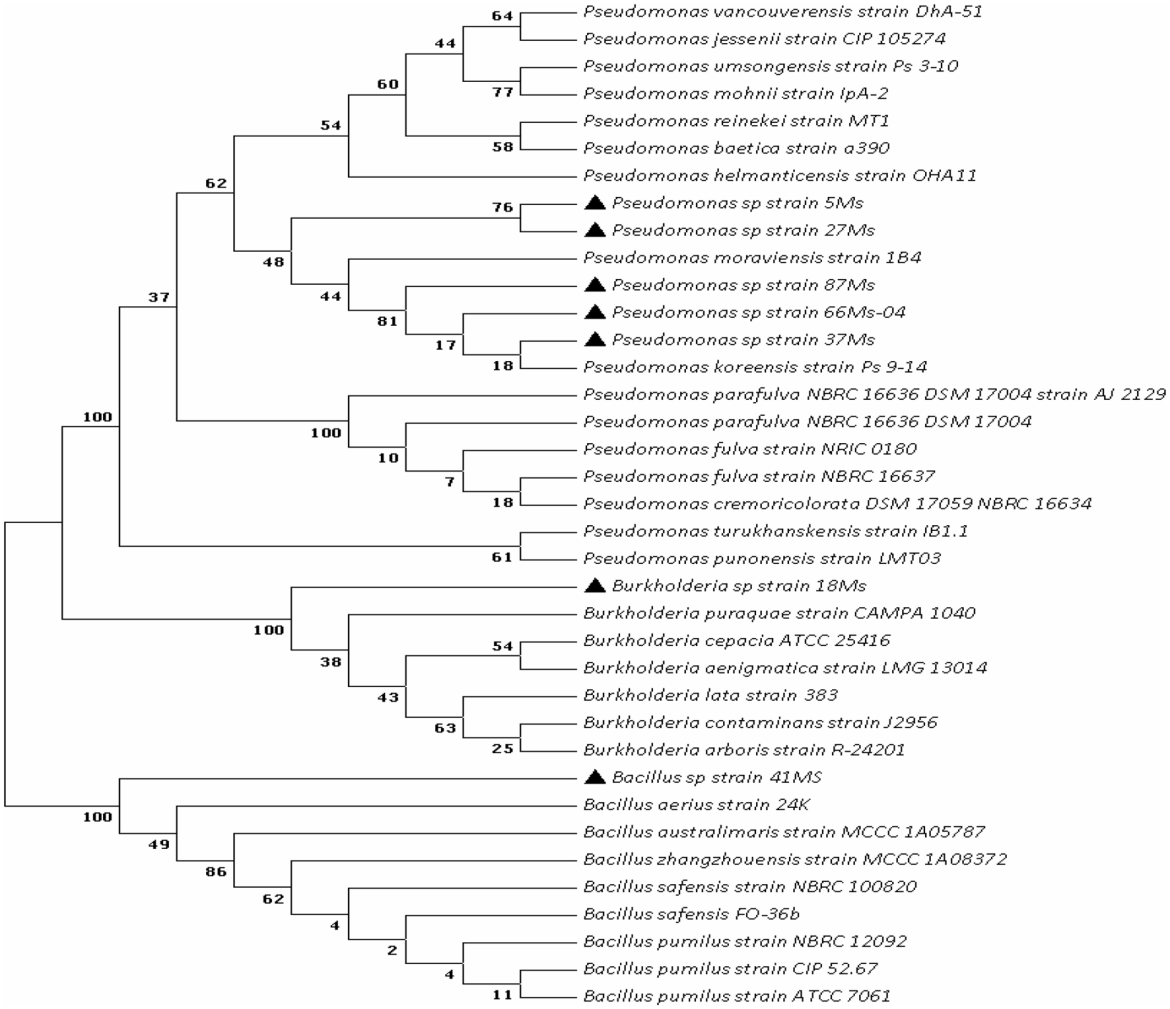
Neighbor-joining phylogenetic tree based on 16S rDNA sequences showing the position of isolated strains compared to some of their closest phylogenetic relatives (the phylogenetic tree produced by Neighbor-joining method with a bootstrap value of 1000).

The isolates were determined via analysis of PCR-amplified 16S rDNA sequences as shown in Table 2. Using the NCBI database, the acquired sequences deposited in Genbank and were compared to the sequences already present in the databases. Most of the obtained strains were affiliated to the *Pseudomonas* sp. Only two strains named 41Ms and 18Ms were affiliated to *Bacillus* sp. and *Burkholderia* sp., respectively (Fig. 1).

### Laboratory bioassays

The mortality of nymphs and adult females of *D. opuntiae* after exposure to different bacterial strains is presented in Tables 2 and 3. Data analysis showed a significant difference (*p* < 0.001) in mortality of *D. opuntiae* nymphs and adult females, caused by the nine bacteria strains at different tested concentrations for various exposure times. Two days after application, 100% mortality of nymphs was recorded for *P. koreensis* strain 66Ms.04, *P. koreensis* strain 37Ms and strain 5Ms at 10^8^ CFU/mL. In 3 days, post-application, *P. koreensis* strain 37Ms and *Pseudomonas* sp. strain 5Ms at 10^8^ (CFU/mL) reached 100% mortality of nymphs, followed by *Pseudomonas* sp. 27Ms (98%) at 10^8^ (CFU/mL). While the lowest percentage mortality (14%) of nymphs was recorded for *Bacillus* sp*.* 41Ms at 10^6^ (CFU/mL) (Table 3). The mortality of nymphs exposed to various exposure times increased noticeably as the tested bacteria’s concentrations increased.

**Table 3:** Mean percentage ± SE of *Dactylopius opuntiae* nymphs’ mortality after exposure to different bacterial strains.

Bacterial strains	Concentrations (CFU/mL)	Mortality (%)		
		1 DAT	2 DAT	3 DAT
*P. koreensis* strain 66Ms-04	10^8^	90 ± 0.00^b^	100 ± 0.00^a^	100 ± 0.00^a^
	10^7^	58 ± 2.00^ef^	66 ± 2.45^e^	76 ± 2.45^c^
	10^6^	40 ± 0.00^gh^	46 ± 2.45^f^	50 ± 0.00^d^
*P. koreensis* strain 37Ms	10^8^	90 ± 0.00^b^	100 ± 0.00^a^	100 ± 0.00^a^
	10^7^	64 ± 2.45^e^	70 ± 0.00^e^	70 ± 0.00^c^
	10^6^	10 ± 0.00^ij^	26 ± 2.45^gh^	30 ± 0.00^efg^
*Pseudomonas* sp*.* 5Ms	10^8^	88 ± 4.40^b^	100 ± 0.00^a^	100 ± 0.00^a^
	10^7^	56 ± 2.45^ef^	68 ± 2.00^e^	70 ± 0.00^c^
	10^6^	40 ± 0.00^h^	44 ± 2.45^f^	50 ± 3.16^d^
*Pseudomonas* sp*.* 27Ms	10^8^	96 ± 2.45^a^	96 ± 2.45^b^	98 ± 2.00^ab^
	10^7^	60 ± 4.47^ef^	66 ± 2.45^e^	70 ± 0.00^c^
	10^6^	14 ± 2.45^ij^	24 ± 2.45^gh^	28 ± 2.00^efg^
*Burkholderia* sp. 18Ms	10^8^	80 ± 3.16^c^	88 ± 2.00^c^	94 ± 4.00^b^
	10^7^	60 ± 0.00^ef^	66 ± 2.45 ^e^	68 ± 2.00^c^
	10^6^	10 ± 0.00^ij^	22 ± 2.00^ghi^	24 ± 2.45^efg^
Strains 38 (unidentified)	10^8^	78 ± 3.74^c^	86 ± 2.45^c^	94 ± 4.00^b^
	10^7^	60 ± 3.16^ef^	70 ± 0.00^e^	70 ± 0.00^c^
	10^6^	10 ± 2.45^ij^	20 ± 0.00^hi^	26 ± 2.45^efg^
*P. koreensis* strain 87Ms	10^8^	72 ± 4.47^cd^	80 ± 0.00^d^	80 ± 0.00^c^
	10^7^	50 ± 0.00^fg^	62 ± 2.00^e^	66 ± 2.45^c^
	10^6^	18 ± 2.00^i^	30 ± 0.00^g^	32 ± 2.00^ef^
*Bacillus* sp*.* 41Ms	10^8^	64 ± 2.45^de^	68 ± 2.00^e^	70 ± 0.00^c^
	10^7^	42 ± 2.00^gh^	46 ± 2.45^f^	48 ± 2.00^d^
	10^6^	10 ± 0.00^j^	14 ± 2.45^i^	14 ± 2.45^i^
Control (Water)		0 ± 0.00^k^	0 ± 0.00^k^	0 ± 0.00^j^

Means in the same column followed by different letter(s) are significantly different based on Tukey test (*p* < 0.05).

*DAT* days after treatment.

**Figure 2:**
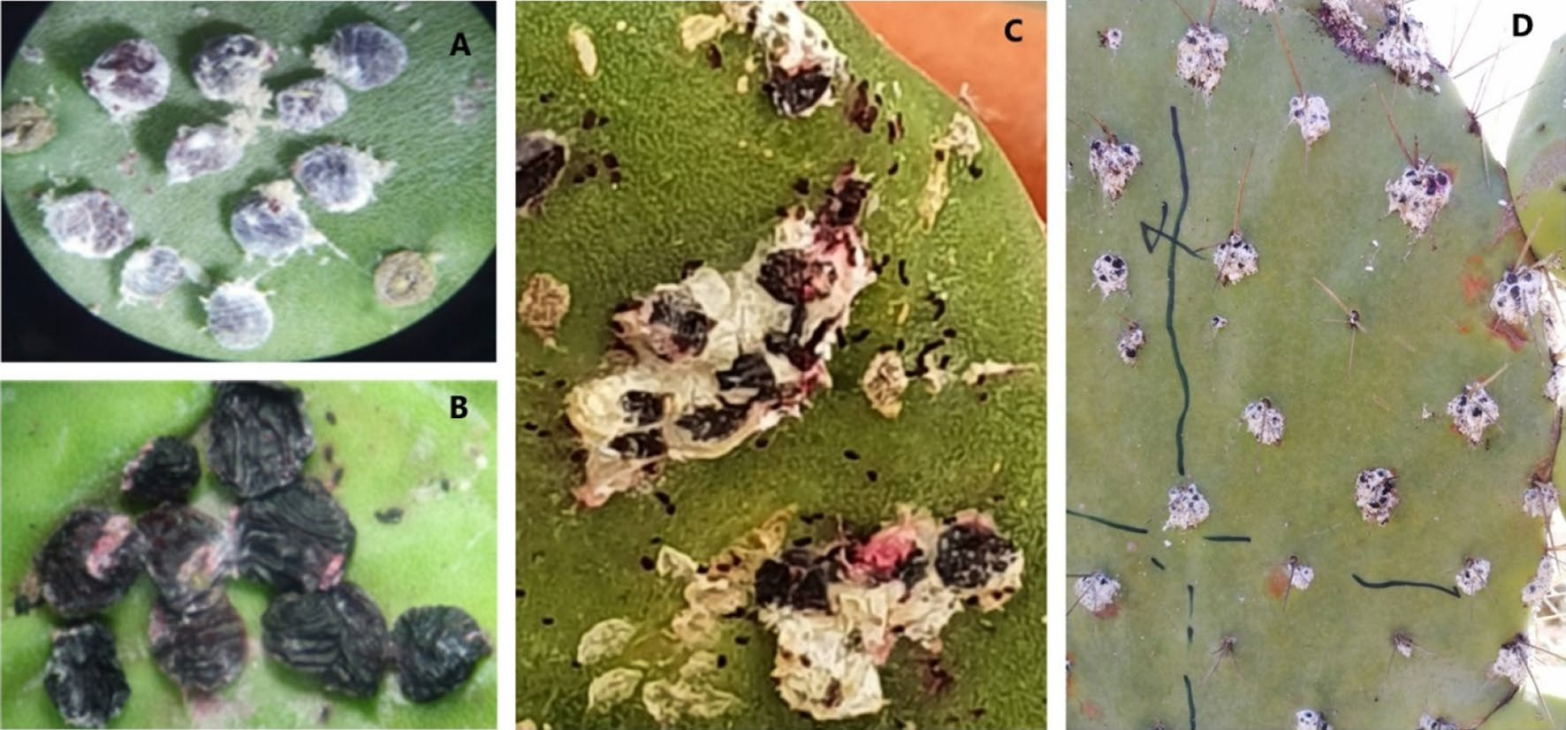
Females of *Dactylopius opuntiae* before (**A**) and after (**B**) application of *Pseudomonas koreensis s*train 66 Ms-04 at (10^8^ CFU/mL) (Laboratory bioassay (G × 40)); in the greenhouse (**C**) and field conditions (**D**).

The results of the statistical data revealed that all the tested bacteria had significantly different mortality rates for adult females at various exposure times (*p* < 0.001; Table 4). The maximum rate of females mortality was recorded for *P. koreensis* strain 66Ms.04 and *Pseudomonas* sp*.* strain 37Ms at 10^8^ (CFU/mL) with 50 and 46%, respectively four days after treatment. Seven days after application, *P. koreensis* strain 66Ms.04 at 10^8^ CFU/mL showed the highest levels of adult females’ mortality (88%) (Fig. 2), followed by both *Pseudomonas* sp. strain 37Ms and *Pseudomonas* sp. strain 5Ms with 72% of adult mortality. However, the lowest mortality was recorded by *Bacillus* sp*.* 41Ms at 10^6^ (CFU/mL) with 6%, 8 days after application (Table 4).

**Table 4:** Mean percentage ± SE of *Dactylopius opuntiae* adult females’ mortality after exposure to different bacterial strains.

Bacterial strains	Concentrations	Mortality (%)					
		3 DAT	4 DAT	5 DAT	6 DAT	7 DAT	8 DAT
*P.koreensis* strain 66Ms-04	10^8^	40 ± 5.48^a^	50 ± 5.48^a^	56 ± 6.78^ab^	74 ± 2.45^a^	88 ± 2.00^a^	88 ± 2.00^a^
	10^7^	6 ± 2.45^de^	14 ± 2.45^cd^	26 ± 4.00^cd^	46 ± 2.45^b^	46 ± 2.45^c^	46 ± 2.45^d^
	10^6^	4 ± 2.45^de^	4 ± 2.45^d^	10 ± 0.00^de^	10 ± 0^ef^	12 ± 2.00^jklm^	20 ± 2.00^fghi^
*P.koreensis* strain 37Ms	10^8^	24 ± 2.45^ab^	46 ± 6.00^a^	68 ± 3.74^a^	70 ± 3.16^a^	72 ± 3.74^b^	72 ± 3.70^bc^
	10^7^	6 ± 2.45^de^	8 ± 2.00^d^	22 ± 3.74^d^	22 ± 3.74^cde^	30 ± 00^defg^	30 ± 0.00^efg^
	10^6^	0 ± 0^e^	6 ± 2.45^d^	10 ± 0.00^de^	16 ± 2.45^def^	16 ± 2.45^ijk^	16 ± 2.45^ghij^
*Pseudomonas* sp*.*5Ms	10^8^	24 ± 2.45^ab^	40 ± 4.47^ab^	56 ± 7.48^ab^	64 ± 5.10^a^	72 ± 2.00^b^	82 ± 5.00^ab^
	10^7^	8 ± 3.74^de^	8 ± 3.74^d^	22 ± 4.90^d^	40 ± 3.16^bc^	40 ± 3.16^cde^	40 ± 3.16^de^
	10^6^	0 ± 0.00^e^	6 ± 4.00^d^	10 ± 3.16^de^	12 ± 2.00^ef^	20 ± 0.00^fghij^	20 ± 4.00^fghi^
*Pseudomonas* sp*.* 27Ms	10^8^	28 ± 10.20^ab^	36 ± 12.88^abc^	46 ± 10.77^bc^	46 ± 10.77^b^	62 ± 2.00^b^	62 ± 2.00^c^
	10^7^	0 ± 0.00^e^	12 ± 2.00^cd^	22 ± 2.00^d^	36 ± 4.00^bc^	36 ± 4.00^cde^	36 ± 4.50^de^
	10^6^	0 ± 0.00^e^	2 ± 2.00^d^	12 ± 2.00^de^	16 ± 4.00^def^	18 ± 3.74^ghij^	18 ± 3.45^ghi^
*Burkholderia* sp. 18Ms	10^8^	2 ± 2.00^e^	4 ± 2.45^d^	16 ± 4.00^de^	30 ± 3.16^bcd^	30 ± 3.16^defg^	30 ± 3.50^defg^
	10^7^	2 ± 2.00^de^	2 ± 2.00^d^	6 ± 2.45^e^	18 ± 3.74^def^	18 ± 3.74^ghi^	18 ± 4.45^ghi^
	10^6^	0 ± 0.00^e^	2 ± 2.00^d^	4 ± 2.45^e^	10 ± 0.00 ^ef^	10 ± 0^ijklm^	10 ± 0.00^ijklm^
Strains 38 (unidentified)	10^8^	12 ± 3.74^bd^	22 ± 6.63^bcd^	30 ± 5.48^d^	30 ± 5.48^bcd^	42 ± 2.00^cd^	42 ± 2.40^de^
	10^7^	0 ± 0.00^e^	10 ± 0.00^cd^	14 ± 2.45^de^	26 ± 2.45^cde^	26 ± 2.45^efghi^	26 ± 2.45^efgh^
	10^6^	0 ± 0.00^e^	2 ± 2.00^d^	12 ± 2.00^de^	14 ± 2.45^def^	14 ± 2.45^ijklm^	14 ± 2.45^hijkl^
*P.koreensis* strain 87Ms	10^8^	26 ± 4.00^ab^	48 ± 3.74^a^	62 ± 2.00^ab^	66 ± 2.45^a^	70 ± 4.47^b^	74 ± 4.47^b^
	10^7^	2 ± 2.00^de^	8 ± 3.74^d^	28 ± 4.90^cd^	30 ± 3.16^bcd^	32 ± 3.74^cdef^	34 ± 4.35^def^
	10^6^	2 ± 2.00^de^	4 ± 2.45^d^	12 ± 2.00^de^	16 ± 2.45^def^	18 ± 2.00^gij^	18 ± 2.00^ghi^
*Bacillus *sp*.* 41Ms	10^8^	2 ± 2.00^e^	2 ± 2.45^d^	12 ± 4.00^de^	22 ± 3.16^cde^	30 ± 3.16^defgh^	30 ± 3.00^defg^
	10^7^	0 ± 0.00^e^	4 ± 2.00^d^	6 ± 2.45^e^	16 ± 3.74^def^	16 ± 3.74^ijkl^	16 ± 3.74^ghijk^
	10^6^	0 ± 0.00^e^	2 ± 2.00^d^	4 ± 2.45^e^	4 ± 0.00^g^	6 ± 0.00^n^	6 ± 1.50^m^
Control (Water)		0 ± 0.00^e^	0 ± 0.00^e^	0 ± 0.00f.	0 ± 0.00^h^	0 ± 0.00^o^	0 ± 0.00^n^

Means in the same column followed by different letter(s) are significantly different based on Tukey test (*p* < 0.05).

### Greenhouse bioassay

**Figure 3:**
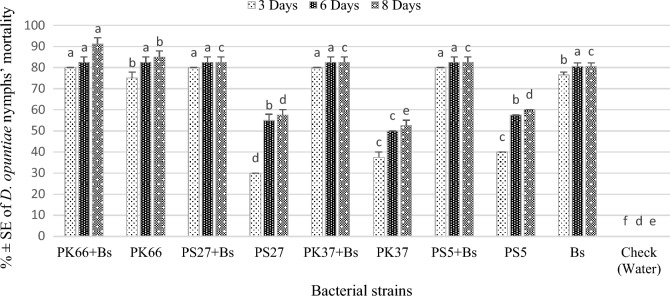
Mean percentage ± SE of *Dactylopius opuntiae* nymphs’ mortality after treatment with various bacterial strains under greenhouse condition. PK66 + Bs: *Pseudomonas koreensis* strain 66Ms-04 + Black soap; PK66: *Pseudomonas koreensis* strain 66Ms-04; PS27 + Bs: *Pseudomonas* sp. 27Ms + Black soap; PS: *Pseudomonas* sp. 27Ms; PK37 + Bs: *Pseudomonas koreensis* strain 37Ms + Black soap; PK37: *Pseudomonas koreensis* strain 37Ms; PS5 + Bs: *Pseudomonas* sp. 5Ms + Black soap; PS5: *Pseudomonas* sp. 5Ms; Bs: Black soap. The different letters indicate significant differences between groups based on Tukey test (*p* < 0.05).

The effects of four bacterial strains on *D. opuntiae* nymphs and adult females' mortality are presented in Tables 4 and 5. The ANOVA showed significant differences in nymphs mortality induced by various treatments and the checks at different exposure times (Fig. 3). Three days after treatments, *P. koreensis* strain 66Ms-04 and *Pseudomonas* sp*.* 27Ms and *P. koreensis* strain 37Ms and 5Ms strains applied at 10^8^ CFU/mL before application of the black soap (60g/L) produced the maximum mortality of nymphs among all tested bacteria, with 80.0% mortality, respectively. For various bacterial strains, nymphs mortality increased with increasing concentrations for various exposure durations. The nymph’s mortalities increased significantly at the 8th day after application to reach its maximum for *P. koreensis* strain 66Ms-04 in combination with black soap (91.25%), followed by *P. koreensis* strain 66Ms-04 applied alone with 85% (Fig. 3).

**Table 5:** Lethal concentrations (LC_50_ and LC_90_) of tested bacterial strains, against *Dactylopius opuntiae* nymphs (at *p* < 0.05) after exposure at different times.

Bacterial strains	Time (Days)	LC50 (LCL–UCL)	LC90(LCL–UCL)	χ^2^	*p*	Intercept	Slope
*P. koreensis* strain 66Ms-04	2	1.79 × 10^6^ (9.24 × 10^5^–2.90 × 10^6^)	3.55 × 10^7^ (1.91 × 10^7^–9.71 × 10^7^)	74.887	0.001^a^	− 3.21	0.52
	3	1.23 × 10^6^ (7.53 × 10^5^–1.79 × 10^6^)	2.15 × 10^7^ (1.39 × 10^7^–3.98 × 10^7^)	38.736	0.001^a^	− 4.29	0.71
*Pseudomonas* sp*.* 5Ms	2	1.87 × 10^6^ (1.09 × 10^6^–2.83 × 10^6^)	3.19 × 10^7^ (1.86 × 10^7^–7.23 × 10^7^)	60.174	0.001^a^	− 3.89	0.62
	3	1.37 × 10^6^ (7.03 × 10^5^–2.21 × 10^6^)	2.97 × 10^7^ (1.67 × 10^7^–7.29 × 10^7^)	61.278	0.001^a^	− 3.15	0.52
*P. koreensis strain* 37 M*s*	2	3.27 × 10^6^ (2.64 × 10^6^–4.01 × 10^6^)	2.65 × 10^7^ (1.97 × 10^7^–3.82 × 10^7^)	25.441	0.021^a^	− 7.71	1.18
	3	2.92 × 10^6^ (2.34 × 10^6^–3.58 × 10^6^)	2.71 × × 10^8^ (2.01 × 10^7^–3.92 × 10^7^)	23.278	0.038^a^	− 6.82	1.05
*Burkholderia* sp. 18 Ms	2	5.10 × 10^6^ (3.85 × 10^6^–6.64 × 10^6^)	1.01 × 10^8^ (6.67 × 10^7^–1.72 × 10^8^)	26.156	0.016^a^	− 6.62	0.99
	3	4.07 × 10^6^ (2.63 × 10^6^–6.00 × 10^6^)	5.44 × 10^7^ (3.16 × 10^7^–1.20 × 10^8^)	70.175	0.000^a^	− 6.32	0.95
*Bacillus* sp. 41 Ms	2	1.98 × 10^7^ (1.43 × 10^7^–2.82 × 10^7^)	9.55 × 10^8^ (4.63 × 10^8^–2.63 × 10^9^)	25.780	0.018^a^	− 5.76	0.79
	3	1.57 × 10^7^ (1.26 × 10^7^–1.97 × 10^7^)	9.76 × 10^8^ (5.80 × 10^8^–1.87 × 10^9^)	12.469	0.490^b^	− 5.24	0.73
Strains *38* (unidentified) Ms	2	5.16 × 10^6^ (3.58 × 10^6^–7.19 × 10^6^)	1.04 × 10^8^ (6.24 × 10^7^–2.10 × 10^8^)	41.792	0.001^a^	− 6.55	0.97
	3	3.64 × 10^6^ (2.32 × 10^6^–5.40 × 10^6^)	5.19 × 10^7^ (3.00 × 10^7^–1.15 × 10^8^)	68.466	0.001^a^	− 6.09	0.92
*P. koreensis* strain 87 Ms	2	4.09 × 10^6^ (3.02 × 10^6^–5.44 × 10^6^)	4.75 × 10^7^ (3.16 × 10^7^–8.16 × 10^7^)	40.603	0.001^a^	− 6.83	1.02
	3	3.20 × 10^6^ (2.40 × 10^6^–4.17 × 10^6^)	3.42 × 10^7^ (2.35 × 10^7^–5.56 × 10^7^)	35.721	0.001^a^	− 6.59	1.01
*Pseudomonas* sp. 27 Ms	2	4.90 × 10^6^ (3.83 × 10^6^–6.17 × 10^6^)	3.58 × 10^8^ (2.25 × 10^8^–6.39 × 10^8^)	7.485	0.875^b^	− 4.57	0.68
	3	3.93 × 10^6^ (2.74 × 10^6^–5.39 × 10^6^)	3.36 × 10^8^ (1.84 × 10^8^–7.61 × 10^8^)	18.286	0.147^a^	− 4.33	0.66

^a^Since goodness-of-fit Chi square is significant (*p* < 0.15), a heterogeneity factor is used.

^b^Since goodness-of-fit Chi square is not significant is (*p* > 0.15), no heterogeneity factor is used in the calculation of confidence limits.

LC_50_ Lethal concentration killing 50% of exposed insects; LC_90_ Lethal concentration killing 90% of exposed insects; LCL 95% lower confidence limits; UCL 95% upper confidence limits; χ^2^ Chi square.

**Figure 4:**
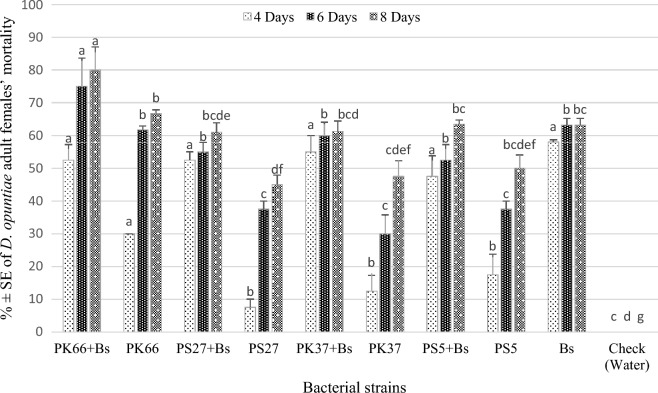
Mean percentage ± SE of *Dactylopius opuntiae* adult females’ mortality after treatment with various bacterial strains under greenhouse condition. PK66 + Bs: *Pseudomonas koreensis* strain 66Ms-04 + Black soap; PK66: *Pseudomonas. koreensis* strain 66Ms-04; PS27 + Bs: *Pseudomonas* sp. 27Ms + Black soap; PS: *Pseudomonas* sp. 27Ms;PK37 + Bs: *P. koreensis* strain 37Ms + Black soap;PK37: *Pseudomonas koreensis* strain 37Ms; PS5 + Bs: *Pseudomonas* sp. 5Ms + Black soap; PS5: *Pseudomonas* sp. 5Ms; Bs: Black soap. The different letters indicate significant differences between groups based on Tukey test (*p* < 0.05).

The ANOVA revealed a significant difference in the mortality of adult females of *D. opuntiae* induced by different bacterial strains and their application with black soap for different exposure periods (*p* < 0.001; Fig. 4). The mortality of adult females for different bacterial isolates increased with increasing concentrations for different exposure times. In 6 days after application, *P. koreensis* strain 66Ms-04 at (10^8^ CFU/mL) combined with black soap (60 g/L) demonstrated the high level of mortality of adult females’ with 75.00%. The highest levels of *D. opuntiae* adult females’ mortality (80%) was recorded by application of *P. koreensis* strain 66Ms-04 (10^8^ CFU/mL) in combination with black soap, 8 days after application, followed by *P. koreensis* strain 66Ms-04 applied alone (66.75%) (Fig. 4).

Probit analysis of the entomopathogenic bacteria effect shows that lethal concentrations (LC) varied between the tested bacterial strains. The estimated LC values of strains against *D. opuntiae* nymphs showed that three days after treatment, *P. koreensis* strain 66Ms-04, recorded LC_50_ = 1.23 × 10^6^ and LC_90_ = 2.15 × 10^7^, followed by *Pseudomonas* sp. 5Ms (LC_50_ = 1.37 × 10^6^ and LC_90_ = 2.97 × 10^7^) (Table 5). While the LC values of strains against *D. opuntiae* females indicated that *P. koreensis* strain 66Ms-04, (LC_50_ = 8.84 × 10^6^ and LC_90_ = 1.70 × 10^8^) were more effective than *Pseudomonas* sp. 5Ms (LC_50_ = 1.23 × 10^7^ and LC_90_ = 3.53 × 10^8^) eight days after treatment (Table 6). On the other hand, when the mortality rates of females were recorded for all four bacterial strains after each exposure time, the LC_50_ values could not be calculated for any of these strains, as none of the concentrations tested resulted in mortality rates higher than 50%.

**Table 6:** Lethal concentrations (LC_50_ and LC_90_) of tested bacterial strains, against *Dactylopius opuntiae* females (at p < 0.05) after exposure at different times.

Bacteria females strains	Days	LC_50_ (LCL–UCL)	LC_90_ (LCL–UCL)	χ^2^	*p*	Intercept	Slope [log10 (dose)]
*P. koreensis* strain 66Ms-04	8	8.84 × 10^6^ (6.44 × 10^6^–1.21 × 10^7^)	1.70 × 10^8^ (1.01 × 10^8^–3.49 × 10^8^)	38.204	0.001^a^	− 7.13	1.03
	7	1.09 × 10^7^ (8.88 × 10^6^–1.35 × 10^7^)	1.35 × 10^8^ (9.56 × 10^7^–2.05 × 10^8^)	21.766	0.059^a^	− 8.39	1.19
	6	1.74 × 10^7^ (1.37 × 10^7^–2.23 × 10^7^)	3.97 × 10^8^ (2.48 × 10^8^–7.20 × 10^8^)	20.260	0.089^a^	− 7.01	0.97
	5	7.98E + 01 (5.97 × 10^7^–2.94 × 10^8^)	2.68 × 10^9^ (7.63 × 10^8^–2.92 × 10^10^)	79.769	0.001^a^	− 5.98	0.73
	4	6.62 × 10^7^ (3.76 × 10^7^–1.50 × 10^8^)	3.87 × 10^9^ (1.05 × 10^9^–1.05 × 10^9^)	54.249	0.001^a^	− 5.61	0.72
*Pseudomonas* sp. 5Ms	8	1.23 × 10^7^ (8.59 × 10^6^–1.78 × 10^7^)	3.53 × 10^8^ (1.81 × 10^8^–9.22 × 10^8^)	40.684	0.001^a^	− 6.33	0.9
	7	1.76 × 10^7^ (1.41 × 10^7^–2.22 × 10^7^)	1.09 × 10^9^ (6.39 × 10^8^–2.11 × 10^9^)	15.003	0.307^b^	− 5.17	0.71
	6	2.89 × 10^7^ (1.88 × 10^7^–4.86 × 10^7^)	1.45 × 10^9^ (5.48 × 10^8^–6.70 × 10^9^)	43.051	0.001^a^	− 5.85	0.78
	5	7.38 × 10^7^ (3.47 × 10^7^–2.71 × 10^8^)	3.97 × 10^9^ (7.59 × 10^8^–1.50 × 10^11^)	103.162	0.001^a^	− 5.39	0.68
	4	2.96 × 10^8^ (8.54 × 10^7^–1.24 × 10^10^)	1.67 × 10^10^ (1.33 × 10^9^–1.24 × 10^14^)	161.074	0.001^a^	− 4.12	0.47
*Pseudomonas* sp. 27 Ms	8	2.02 × 10^7^ (1.22 × 10^7^–3.65 × 10^7^)	8.46 × 10^8^ (3.01 × 10^8^8.02 × 10^9^)	67.311	0.001^a^	− 5.88	0.81
	7	2.54 × 10^7^ (1.59 × 10^7^–4.50 × 10^7^)	1.43 × 10^9^ (4.96 × 10^8^–8.02 × 10^9^)	49.941	0.001^a^	− 5.44	0.74
	6	3.35 × 10^7^ (2.22 × 10^7^–5.55 × 10^7^)	2.04 × 10^9^ (7.73 × 10^8^–8.92 × 10^9^)	34.065	0.001^a^	− 5.4	0.72
	5	4.48 × 10^7^ (2.91 × 10^7^–7.79 × 10^7^)	2.24 × 10^9^ (8.26 × 10^8^–1.04 × 10^10^)	37.973	0.001^a^	− 5.76	0.75
	4	1.37 × 10^8^ (6.42 × 10^7^–5.76 × 10^8^)	2.87 × 10^9^ (6.50 × 10^8^–9.13 × 10^10^)	125.565	0.001^a^	− 5.62	0.68
*P. koreensis* strain 37 Ms	8	2.54 × 10^7^ (1.70 × 10^7^–4.05 × 10^7^)	9.86 × 10^8^ (4.17 × 10^8^–3.61 × 10^9^)	42.087	0.001^a^	− 5.97	0.81
	7	2.54 × 10^7^ (1.70 × 10^7^–4.05 × 10^7^)	9.86 × 10^8^ (4.17 × 10^8^–3.61 × 10^9^)	42.087	0.001^a^	− 5.97	0.81
	6	3.46 × 10^7^ (1.95 × 10^7^–7.55 × 10^7^)	1.43 × 10^9^ (4.25 × 10^8^–1.33 × 10^10^)	82.124	0.001^a^	− 5.86	0.78
	5	3.97 × 10^7^ (2.66 × 10^7^–6.49 × 10^7^)	9.90 × 10^8^ (4.36 × 10^8^–3.41 × 10^9^)	47.262	0.001^a^	− 6.7	0.88
	4	1.77 × 10^8^ (7.42 × 10^7^–1.02 × 10^9^)	6.30 × 10^9^ (1.07 × 10^9^–4.24 × 10^11^)	111.040	0.001^a^	− 5.37	0.64

^a^Since goodness-of-fit Chi square is significant (*p* < 0.15), a heterogeneity factor is used.

^b^Since goodness-of-fit Chi square is not significant is (*p* > 0.15), no heterogeneity factor is used in the calculation of confidence limits.

LC_50_ Lethal concentration killing 50% of exposed insects; LC_90_ Lethal concentration killing 90% of exposed insects; LCL 95% lower confidence limits; UCL 95% upper confidence limits; χ^2^ Chi square.

### Field bioassays

The mortality of *D. opuntiae* nymphs and adult females after exposure to the most effective bacterial strain is presented in Table 7. The statistical analysis using ANOVA revealed a significant difference in mortality rates of nymphs and females of *D. opuntiae* when exposed to *P. koreensis* strain 66Ms-04 and when used before the application of black soap for varying durations in the first and second treatment under field conditions (*p* < 0.01; Table 7). On the 3rd days after the first treatments, *P. koreensis* strain 66Ms-04 at 1 × 10^8^ CFU/mL in combination black soap (30 g/L) and black soap applied alone showed the highest mortality rates of nymphs with 80 and 76.66%, respectively. Seven days after the first treatments, the mortality rates did not change significantly for both treatments, except for *P. koreensis* strain 66Ms-04 applied alone that reached 20% mortality. Five days after the second application, the nymph mortality increased to 93.33% for *P. koreensis* strain 66Ms-04 before treatment with black soap (60 g/L). However, the application of this bacterial strain alone reached only 50% mortality.

**Table 7:** Insecticidal effects ± SE of *Pseudomonas koreensis* strain Ps 9–14 and their combination with black soap on *Dactylopius opuntiae* nymphs and adult females under field condition.

Treatments/Exposure Period	(1st spray)			(2nd spray)		
	3 DAT	5 DAT	7 DAT	3 DAT	5 DAT	7 DAT
Mortality (%) against nymphs						
*P. koreensis* strain 66Ms-04 + Black soap	80 ± 3.00^a^	83.33 ± 3.33^a^	83.33 ± 3.33^a^	90 ± 4.50^a^	93.33 ± 3.33^a^	93.33 ± 3.33^a^
*P. koreensis* strain 66Ms-04	3.33 ± 3.33^b^	20 ± 2.00^b^	20 ± 2.00^b^	50 ± 3.00^b^	50 ± 4.50^b^	50 ± 3.00^b^
Black soap	76.67 ± 3.33^a^	80 ± 3.00^a^	80 ± 3.00^a^	90 ± 4.50^a^	90 ± 4.50^a^	90 ± 4.50^a^
Check (Water)	0 ± 0.00^b^	0 ± 0.00^c^	0 ± 0.00^d^	0 ± 0.00^d^	0 ± 0.00^d^	0 ± 0.00^d^
Mortality (%) against adult females						
*P. koreensis* strain 66Ms-04 + Black soap	27.77 ± 4.00^a^	48.33 ± 1.67^a^	60 ± 3.00^a^	70 ± 3.50^a^	75 ± 3.50^a^	78 ± 3.60^a^
*P. koreensis* strain 66Ms-04	3.33 ± 3.33^c^	15 ± 2.89^c^	15 ± 2.89^c^	16.67 ± 1.67^c^	16.67 ± 1.67^c^	16.67 ± 1.67^c^
Black soap	14.33 ± 2.33^b^	27.67 ± 1.45^b^	31 ± 1.00^b^	33.33 ± 1.67^b^	33.33 ± 1.67^b^	35 ± 2.89^b^
Check (Water)	0 ± 0.00^c^	0 ± 0.00^d^	0 ± 0.00^d^	0 ± 0.00^d^	0 ± 0.00^d^	0 ± 0.00^d^

Means in the same column followed by different letter(s) are significantly different based on Tukey test (*p* < 0.05).

The ANOVA analysis indicated a statistically significant difference in the mortality of females of *D. opuntiae* produced by different treatments, performed for varying exposure times during the first and second treatment (*p* < 0.01; Table 7). The mortality rate of female adults increased to 60%, 7 days after the first treatment by *P. koreensis* strain 66Ms-04 (10^8^ CFU/mL) applied with black soap. Seven days after the second application of *P. koreensis* strain 66Ms-04 applied with black soap, the mortality rate of adult females considerably increased to reach 78%.

## Discussion

Plant growth promoting (PGP) bacteria are well known for their usefulness in crop production and protection and in maintaining soil quality. In the local context of Morocco, the use of local antagonistic PGP bacteria including *Bacillus* spp., *Pseudomonas* spp., have been cited not only to improve plant growth, but also as a possible eco-friendly alternative to control insect pests and plant pathogens^28, 29^. In the present study, we evaluated the insecticidal potential of various bacterial strains applied alone and combined with a detergent for the control of *D. opuntiae* nymphs and adult females. Among the different microbial strains tested to control *D. opuntiae* at various stages, the best results were achieved with a double application of *P. koreensis* strain 66Ms-04 at a concentration of 10^8^ CFU/mL, in combination with black soap at a concentration of 60 g/L. The results showed that the insecticidal activity of different bacterial strains was found to increase with increasing concentrations and exposure times under laboratory conditions. However, the insecticidal effect of the *P. koreensis* strains exhibited greater efficacy when used in combination with black soap, without causing any noticeable harm to the treated plants. Many authors have identified more than 100 bacterial species with entomopathogenic activity^18, 30–32^.

Among the genera of entomopathogenic bacteria most used in the management of various insect pests, we found *Bacillus*, *Pseudomonas*, *Lysinibacillus*, *Serratia* and *Chromobacterium*, *Xenorhabdus*, *Photorhabdus*^33–35^. Among the bacteria used for commercial purposes, *L. sphaericus, B. popilliae*, *C. bifermentans, B. thuringiensis*, *P. alcaligenes*, *S. spinosa, Pseudomonas aureofaciens*, *S. avermitilis* and *S. entomophila* were considered the most used and appreciated microbial pest control agents.

The genus *Pseudomonas* belongs to the Gammaproteobacteria, a class of bacteria that emerged from the *Hydrobacteria* 1.75 billion years ago^36^, and belonging to the family of Pseudomonadaceae which has been studied extensively and has over 200 described species^37^. *Pseudomonas* is one of the most ubiquitous genera in the world; they are founded in environmental habitats such as the soil^38^, the surface of plants^39, 40^and the guts of insects^41^. These bacteria are very adaptive and capable to use a large range of compounds as an energy source. There are many species that occur in association with plants and animals, mainly as saprophytes, but some are also pathogenic to them^42^.

*Pseudomonas* bacteria have beneficial applications in biotechnology, in the promotion, of plant growth, in bioremediation and in biological control^43^. Species of pseudomonads that are pathogenic to insects include, *Pseudomonas aeruginosa*, *Pseudomonas protegens, Pseudomonas chlororaphis*, *Pseudomonas fuorescens*, *Pseudomonas putida*, *Pseudomonas entomophila*, *Pseudomonas taiwanensis*, *Pseudomonas mosselli*, *Pseudomonas syringae*, and several more strains of *Pseudomonas* spp.^44–53^.

The insecticidal properties of *P. koreensis* have been documented in various studies, indicating its ability to effectively control a broad spectrum of insect pests. *P. koreensis* is a gram-negative bacteria, first described as a new species by Kwon et al.^54^. The species was obtained from a Korean agricultural soil with low pH and can to grow at 4 °C.

Hultberg et al.^55^ showed that *P. koreensis* 2.74 (CBS 125413) produces the Cyclic lipopeptides (CLP) lokisin and a crude extract of this CLP with a protective effect against tomato disease *Pythium ultimum*. The same search also revealed that *P. koreensis* 2.74 and the CLP significantly reduce potato late blight disease induced by *Phytophthora infestans* in a detached-leaf test^56^.

Ruffner et al.^48^ demonstrated that Fit toxin producing *Pseudomonas* exhibit potent oral activity against larvae of *Spodoptera littoralis* (Lepidoptera: Noctuidae), *Chloridea virescens* (Lepidoptera: Noctuidae) and *Plutella xylostella* (Lepidoptera: Plutellidae). Spraying plant leaves with suspensions containing only 1000 *Pseudomonas* cells per ml was sufficient to kill 70–80% of *Spodoptera* and *Helicoverpa* larvae.

In addition, the study of Rangel et al.^57^ showed that the three strains within the *P. chlororaphis* subgroup exhibited both oral and injectable toxicity to the tobacco hornworm *Manduca sexta* (Lepidoptera: Sphingidae). The three strains possess the gene cluster encoding for the insect toxin FitD. The same authors reported that *P. protegens* Pf-5 exhibited substantial levels of oral toxicity against the dipteran *Drosophila melanogaster* (Diptera: Drosophilae)^57^. A number of *P. fluorescens* strains and *P. protegens* Pf-5 have been shown to kill or to cause morphologic defects in *D. melanogaster* adult flies that emerged from the infected larvae^57, 58^.

In the present study, the black soap detergent at 60 g/L was employed to remove the thicker wax, making female and nymph *D. opuntiae* more susceptible to the strong contact toxicity of the bacterial strain tested. Black soap is a natural product produced from fatty acids obtained from olive oil. Secondary metabolite production in *Pseudomonas* has been reviewed extensively. It has become evident that only a limited number of bioactive compounds play a clear role in biocontrol of plant diseases such as hydrogen cyanide (HCN), 2,4-diacetylphloroglucinol (DAPG); phenazines, pyrrolnitrin, pyoluteorin, 2-hexyl-5-propyl-alkylresorcinol, siderophores; and (cyclic) lipopeptides^42^. In addition, Lin et al.^59^ reported that *P. koreensis* CRS05-R5 exhibited a biocontrol effect against *Sitophilus oryzae* (Coleoptera: Curculionidae) and *Acidovorax avenae* subsp. *avenae*. The study of Ichikawa et al.^60^ showed that *P. koreensis* CRS05-R5 genome had more than 800 genes predicted to be involved in secondary metabolism. The present study showed that both *Bacillus* species (*Bacillus* sp. 41Ms and *B. thuringiensis* subsp. *kurstaki* ABTS-351) did not show a good efficacy to control the mature females. However, the *Bacillus* sp. 41Ms species resulted in moderate mortality against nymphs 3 days after application. In contrast, Idris et al.^15^ reported a significant insecticidal effect against both nymphs and females of *D. opuntiae* using the crude enzyme solution produced by *Bacillus subtilis* SY134D strain at concentration 100%. This strain of *B. subtilis* SY134D used reported as a good producer of chitinase and lipase and other six hydrolytic enzymes. The author suggests that the death of nymphs and adults mature could be attributed to cochineal wax hemolysis by the lipase and then chitin degradation by chitinase.

## Conclusions

The results of this study suggest that the combination of double applications of the bacterial strain *P. koreensis* 66Ms-04 at 10^8^ CFU/mL with black soap at 60 g/L could be used as a component of integrated pest management (IPM) for controlling *D. opuntiae*. This approach provides an effective and environmentally friendly alternative to chemical insecticides. However, further research is necessary to understand the mechanisms and identify the causes of toxicity against different stages of *D. opuntiae*, by identifying the responsible bacterial metabolites, enzymes, their combinations. Through this process, researchers may be able to identify the most efficient and targeted way to use these bacteria to manage the cochineal insect.

The interaction of these pseudomonads with other biocontrol agents, which could have a synergistic effect on wild cochineal control, can also be studied. These findings showed that entomopathogenic bacteria are promising for developing a biopesticide formulation for the control of *D. opuntiae* as an effective and safe alternative to pesticides.

### Patents

One patent resulting from the work reported in this manuscript. The patent titled: Bacterial strain of *Pseudomonas koreensis* and an insecticide composition for the control of the wild cochineal *Dactylopius opuntiae*. Patent registered in the Moroccan office of industrial and commercial property (OMPIC) under number 5794.

## Data Availability

The data is available on request from the corresponding author (KEL).
